# An Extracellular Matrix Molecule, Secreted by the Epithelial-Mesenchymal Transition is Associated With Lymph Node Metastasis of Thyroid Papillary Carcinoma

**DOI:** 10.5812/ijem.10748

**Published:** 2014-01-01

**Authors:** Hiroshi Takeyama, Yoshinobu Manome, Kouki Fujioka, Isao Tabei, Hiroko Nogi, Yasuo Toriumi, Kumiko Kato, Makiko Kamio, Yoshimi Imawari, Satoki Kinoshita, Naoshi Akiba, Ken Uchida, Toshiaki Morikawa

**Affiliations:** 1 Department of Surgery, Jikei University School of Medicine, Tokyo, Japan; 2 Department of Molecular Cell Biology, Jikei University School of Medicine, Tokyo, Japan

**Keywords:** Extracellular Matrix, Epithelial-Mesenchymal Transition

## Abstract

**Background:**

Papillary thyroid carcinoma often has lymph node metastasis, compared with follicular thyroid carcinoma. The study showed that epithelial-mesenchymal transition occurs in carcinoma cells during the first stage of metastasis, where some extracellular matrix molecules are secreted in large quantities. Sialic acid carried by fibronectin as the antigen of the monoclonal antibody (MoAb) JT-95, was detected in 90% of papillary thyroid carcinoma cases, and in a few follicular thyroid carcinomas, in the extracellular matrix of thyroid carcinoma cells.

**Objectives:**

The current study was conducted to investigate the association between increasing the number of extracellular matrix molecules, fibronectin, and lymph node metastasis. We also co-cultured a thyroid carcinoma cell line and lymphocyte cell line, with and without MoAb JT-95, in order to investigate the mechanism of cell to cell interaction.

**Patients and Methods:**

Immunostaining with JT-95 was performed in 45 papillary thyroid carcinoma cases, and 20 follicular type tumors, to investigate the association between the quantity of fibronectin expression and the frequency of lymph node metastasis. The thyroid carcinoma cell line (SW1736), which secreted fibronectin, and the B cell-lymphoma cell line (Daudi), which held integrin on the cell surface, were co-cultured to observe the adhesion of cells to each other. The SW1736 cell line, pretreated with JT-95, was also co-cultured with the Daudi cell line.

**Results:**

There were 39 cases with lymph node metastasis in 59 malignant tumors, and 0 cases in 6 benign follicular type tumors. The staining scores by JT-95 of the 39 tumors with lymph node metastasis were 5+ in eight cases and 6+ in 31 cases. On the other hand, the scores of 20 malignant tumors without lymph node metastasis were < 4+ in all of the cases. In the co-cultured assay, numerous adhesions were observed between the SW1736 and Daudi cells. In contrast, the inhibition of adherences was observed in proportion to the concentrations of JT-95.

**Conclusions:**

Increased fibronectin expression in thyroid malignancies is correlated with lymph node metastasis.

## 1. Background

Differentiated carcinomas, known as papillary and follicular carcinomas, are the most common malignancies of the thyroid, and often diagnosed with a physical examination. Among these malignancies, the rate of correct preoperative diagnosis of papillary thyroid carcinoma (PTC) is greater than 90% using a physical examination, such as; a fine needle aspiration (FNA) biopsy, ultrasound examination (US), or thallium-technetium (Tl-Tc) scintigram. On the other hand, follicular thyroid carcinoma (FTC) has a lower rate of correct diagnosis as 20% to 42%, because it is histologically and morphologically difficult to differentiate between this tumor and benign follicular tumors (1-3). Moreover, the recurrence or metastasis pattern is different between PTC and FTC. PTC often has lymph node metastasis in 80% of operated cases, compared to 7-10% of FTC cases. However, the rate of remote metastasis, such as lung or bone via the blood stream, is higher in FTC than PTC (4-6). Thus, many studies have attempted to find differences between PTC and FTC. Some studies have found that oncofetal fibronectin (OnfFN), which leads to the isoform of the III connecting segment (III-CS), or it is alternatively spliced from the fibronectin (FN) during tumorigenesis, is detected in most PTC and FTC cases (7-9). A monoclonal antibody (MoAb), designated JT-95, was raised against the membrane fraction of PTC, as well as recognized as a glycochain containing sialic acid, carried by sialyl fibronectin (sFN) as antigens. 

Antigens of MoAb JT-95 were detected in more than 90% of PTC cases, and in 10% -15% of FTC cases in our series (10, 11).

## 2. Objectives

The aim of this study was to investigate the association between the sFN expression quantity and the frequency of lymph node metastasis in thyroid malignancies, by performing MoAb JT-95 staining in PTC and FTC cases, including follicular type tumors and lymph node metastasis regions. Moreover, to investigate the mechanism of interaction, we co-cultured thyroid carcinoma cell lines producing sFN, and lymphocyte cell lines which held integrin on the cell surface as a receptor of sFN. We also co-cultured the cell lines with MoAb JT-95 to observe potential changing of cells adhesion 

## 3. Patients and Methods

### 3.1. Patients

Twenty FTC tumor patients (9 males and 11 females) and 45 PTC patients (9 males and 36 females), operated at the Jikei University, Tokyo, in 2009, were examined. At the time of operation, their ages ranged from 24 to 86 years, with a mean age of 47, and a median age of 42 years. Twenty FTC tumor cases were suspected of being malignant based on one or more positive results from preoperative examinations involving; ultrasonography (US), Tl-Tc scintigraphy, or fine needle aspiration (FNA) cytology. These tumors were solid and isolated, with a size greater than 40 mm. The PTC patients were also examined preoperatively. The results of FNA in 6 PTC cases revealed class IV and 39 cases as class V, and the tumors revealed solid, hypoechoic, irregular masses with microcalcifications by US. The tumors were suspected carcinomas.

### 3.2. Methods

#### 3.2.1. MoAb JT-95 Preparation and Immunohistochemical Staining

The production and characterization of MoAb JT-95 was as previously described (10). Briefly, mice were immunized with a soluble membrane extract of human PTC, and the isotype JT-95 was IgM.JT-95 recognized as a glycochain containing sialic acid, carried by both FN and gangliosides as antigens (11). The immunoreactivity of MoAb JT-95 was tested on human tissues fixed and embedded in paraffin. Tissue sections (3μm) were incubated with 3% H_2_O_2_ in methanol to block endogenous peroxidase for 30 minutes. Sections were saturated with 10% normal horse serum for 20 minutes at room temperature. The tumor and lymph nodes, containing metastasis sections, were stained with 1 μg/mLMoAb JT-95 using a Vectastain kit (Vector Laboratories, Burlingame, CA, The USA) for the immunohistochemical study.

#### 3.2.2. Data Interpretation

##### 3.2.2.1. Pathological Diagnosis

The final tumor diagnosis was determined by hematoxylin-eosin (H&E) stained histological sections.

##### 3.2.2.2. MoAb JT-95 Staining

As sFN presents in the extracellular matrix (ECM) of the cells, cell localization was classified into three categories: (a) membranous only; (b) membranous and cytoplasmic (heterogeneous); and (c) cytoplasmic staining only. If the tumor cells were stained at the membrane or heterogeneous, the cells were considered positive for MoAb JT-95. The absence of staining or cytoplasmic staining was considered as a negative result and given a score of (0+). Positive specimens were evaluated further for cell intensity and the range of the staining area by two pathologists and one physician. One physician means one surgeon, The staining intensity was classified as weak (1+), moderate (2+), and strong (3+). The staining range of the entire set of tumors was also scored from (1+) to (3+). When less than 10% of cells in the tumor were stained, the range score allocated was (1+), and when 10% -50% of the tumor cells were positive, the score was (2+). If there were more than 50% of the tumor cells stained, the range score allocated was (3+). The tumor was evaluated using combined intensity and range scores. After scoring, we selected the middle score between the three evaluations to be the patient's MoAb JT-95 score.

#### 3.2.3. Cell Line Selection and Co-culture

Several cell lines of thyroid carcinoma were cultured on glass, fixed with 5% paraformaldehyde, and stained with 1μg/mLMoAb JT-95, to confirm the existence of sFN in the ECM. Several malignant lymphoma cell lines were cultured with 1mg/ mL of anti-integrin antibodies (alpha 4+ beta 1), named P4C2 (Abcam, Japan), and flow cytometry was performed to detect integrin on the cell surface. SW1736 human thyroid carcinoma cells (provided by the Memorial Sloan-Kettering Cancer Center, New York, NY) producing sFN, were cultivated with GIBCOTM Leibovitz's L-15 medium (Invitrogen) supplemented with 10% fetal bovine serum. Lymphocytic lymphoma Daudi cells (ATCC, Rockville, MD) expressing integrin, were cultivated with Dulbecco’s minimal essential medium, supplemented with 10% fetal bovine serum. Next, 1x105 of SW1736 cells were seeded in 6 well bottom plates for 12 hours before the experiment. These cells were incubated with 0 mg/mL, 0.2 mg/mL and 1 mg/mL of JT95 monoclonal antibody for 6 hours. After the treatment, 1x105 of Daudi cells were added onto the plate and incubated for 24 hours at 37°C. The cells were washed twice with phosphate buffered saline. As the Daudi were floating cells, un-attached Daudi cells were washed away, the cells on the plate were observed using a phase contrast microscope. Combined Daudi cells with SW1736 cells were also counted in the bottom of each of the 6 wells, and then averaged.

### 3.3. Statistical Analysis

The statistical difference of the staining score of JT-95 between the benign tumors and malignant tumors was calculated using a Mann-Whitney U test as a non-parametric method. The statistical difference between the tumors with lymph node metastasis and without metastasis was also calculated (n =65).

## 4. Results

### 4.1. Final Pathological Diagnosis of Tumors

The final diagnoses of the 20 follicular type tumors were FTC in six cases, follicular variant of papillary thyroid carcinoma (FVPTC) in eight cases, follicular adenoma (FA) in five cases, and adenomatous goiter (AG) in one case (Table 1).

Forty five tumors were diagnosed as PTC (Table 2). 

**Table 1.** Association Between Lymph Node Metastasis and JT-95 Staining in Follicular Type Tumors

Pathological Diagnosis of Follicular Type Tumor	No. Regional Lymph Node Metastasis	Range and Intensity Score of JT-95 Staining		
		Range	Intensity	Total
**FTC^a^**	0/2	1+	2+	3+
	0/1	1+	3+	4+
	1/12	2+	3+	5+
	0/1	0+	0+	0+
	0/1	0+	0+	0+
	0/1	1+	1+	2+
**FVPTC** ^**a**^	0/3	1+	3+	4+
	16/18	2+	3+	5+
	0/2	1+	1+	2+
	0/4	1+	2+	3+
	2/9	2+	3+	5+
	0/2	1+	2+	3+
	2/3	3+	3+	6+
	0/1	0+	0+	0+
**FA** ^**a**^	0/2	1+	1+	2+
	0/2	0+	0+	0+
	0/2	2+	1+	3+
	0/4	0+	0+	0+
	0/1	0+	0+	0+
**AG** ^**a**^	0/2	2+	1+	3+

^a^Abbreviations: AG, adenomatous goiter; FA, follicular adenoma; FTC, follicular thyroid carcinoma; FVPTC, follicular variant of papillary thyroid carcinoma.

**Table 2.** Status of JT-95 Staining in 45 Papillary Carcinoma Cases

Pathological Diagnosis	Range, Intensity Score of JT-95 Staining and LNM^a^ Cases		
	Total Score	Cases	LNM Cases
**Papillary thyroid carcinoma (n = 45)**	0	0	0
	1+	0	0
	2+	3	0
	3+	7	0
	4+	0	0
	5+	5	5
	6+	30	30

^a^ Abbreviations: LNM, lymph node metastasis.

### 4.2. MoAb JT-95 Immunohistochemical Staining

**Figure 1.**
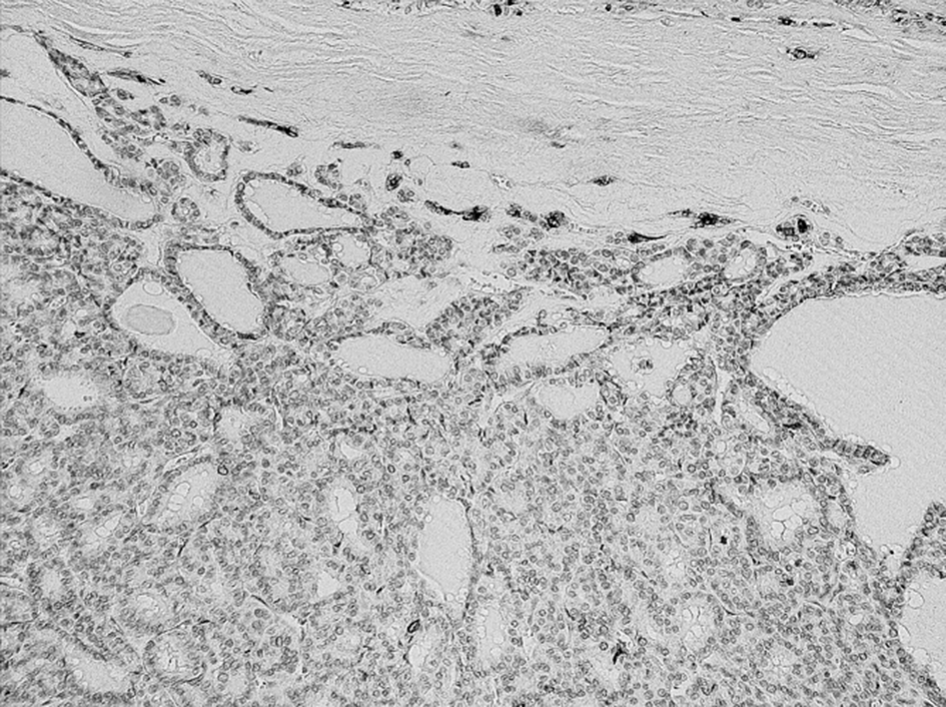
Expressions of sFN in Follicular Thyroid Carcinoma Magnification x 20

The results of the range, intensity, and combined total staining scores of 20 follicular type tumors are summarized in Table 1. The total staining scores ranged from 0+ to 6+ in the malignant lesions. The scores were: 0+ in 2, 2+ in 1, 3+ in 1, 4+ in 1, and 5+ in 1 of the six patients with FTC (Figure 1) (Table 1). 

**Figure 2.**
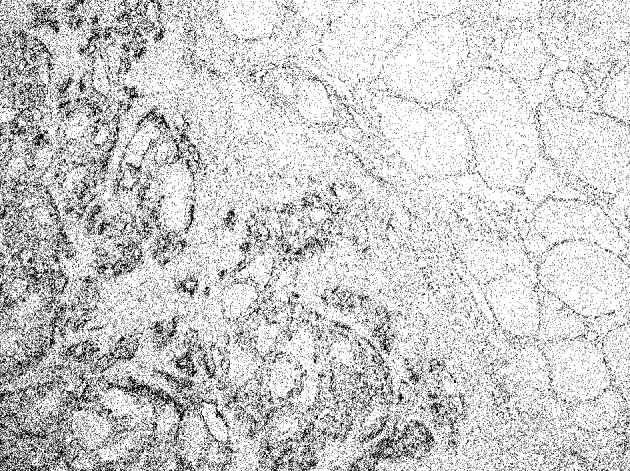
Expressions of sFN in Papillary Thyroid Carcinoma Magnification x 20

The scores were: 0+ in 1, 2+ in 1, 3+ in 2, 4+ in 1, and 5+ in 2, 6+ in 1 of the eight patients with FFVPTC (Table 1). Among the 45 patients with PTC, the score was 2+ in 3, 3+ in 7, 5+ in 5, and 6+ in 30, and no staining in the normal thyroid cells (Figure 2) (Table 2). 

**Figure 3.**
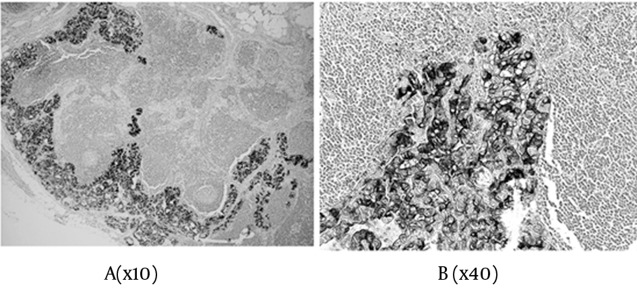
Expressions of sFN in Lymph Node Metastasis A) Magnification x10; B) Magnification x40

In total, the scores were: 0+ in three cases, 2+ in five cases, 3+ in ten cases, 4+ in two cases, 5+ in eight cases, and 6+ in 31 cases, of the 59 malignant tumors (Table 1 & 2). Concerning the metastatic regions in the lymph nodes, the total staining score was 5+ in 11 cases, and 6+ in 28 cases (Figure 3). There were 35 PTC (35/45), 1 FTC (1/6), and 3 PVPTC (3/8), with lymph node metastasis in malignant tumors (Table 1 & 2). In the benign lesions, the score was 0+ in three cases, 2+ in one case, and 3+ in one of the five patients with FA, and 3+ in the one patient with AG. Finally, the scores were 0+ in three cases, 2+ in one case, and 3+ in two cases, of the six benign tumors (Table 1). 

### 4.3. Association Between JT-95 Staining Score and Lymph Nodes Metastasis

There were 39 cases with LNM in 59 malignant tumors, and 0 cases in six benign follicular type tumors. The staining scores by JT-95 of the 39 tumors with LNM were 5+ in eight cases, and 6+ in 31 cases. The score of 5+ cases were: one in FTC, two in FVPTC, and five in PTC. The score of 6+ cases were: one in FVPTC, and 30 in PTC. Thus, the JT-95 scores for all 39 malignant tumors with LNM were ≥ 5+ (Tables 1 & 2). On the other hand, the JT-95 scores of 20 malignant tumors without LNM were 0+ in three cases, 2+ in five cases, 3+ in ten cases, and 4+ in two cases.

### 4.4. Statistical Analysis

Regarding tumor staining, a statistical difference was not suggested in the association of JT-95 staining scores between 59 malignant tumors and six benign follicular type tumors, when the staining scores of ≥ 4+ were considered as positive proof in the JT-95 test. On the other hand, according to the presence or absence of lymph node metastasis in the 59 malignant tumor cases, a statistical analysis revealed a significant difference between patients with JT-95 staining scores of ≥ 4+ and those with lower scores (χ^^2^calibration, P < 0.01).

### 4.5. Association Between Co-cultured Cell Lines

**Figure 4.**
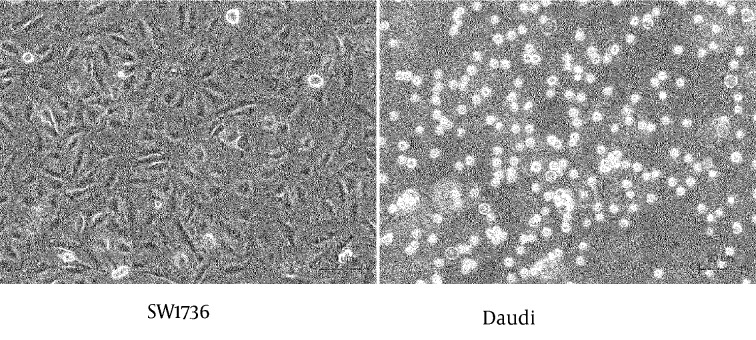
SW1736 Cell and Daudi Cells Before Co-culture

The SW1736 producing sFN, and Daudi expressing integrins, were co-cultured with or without MoAb JT-95. Cells cultured individually in three different areas of the culture plate, are shown in Figure 4. 

**Figure 5.**
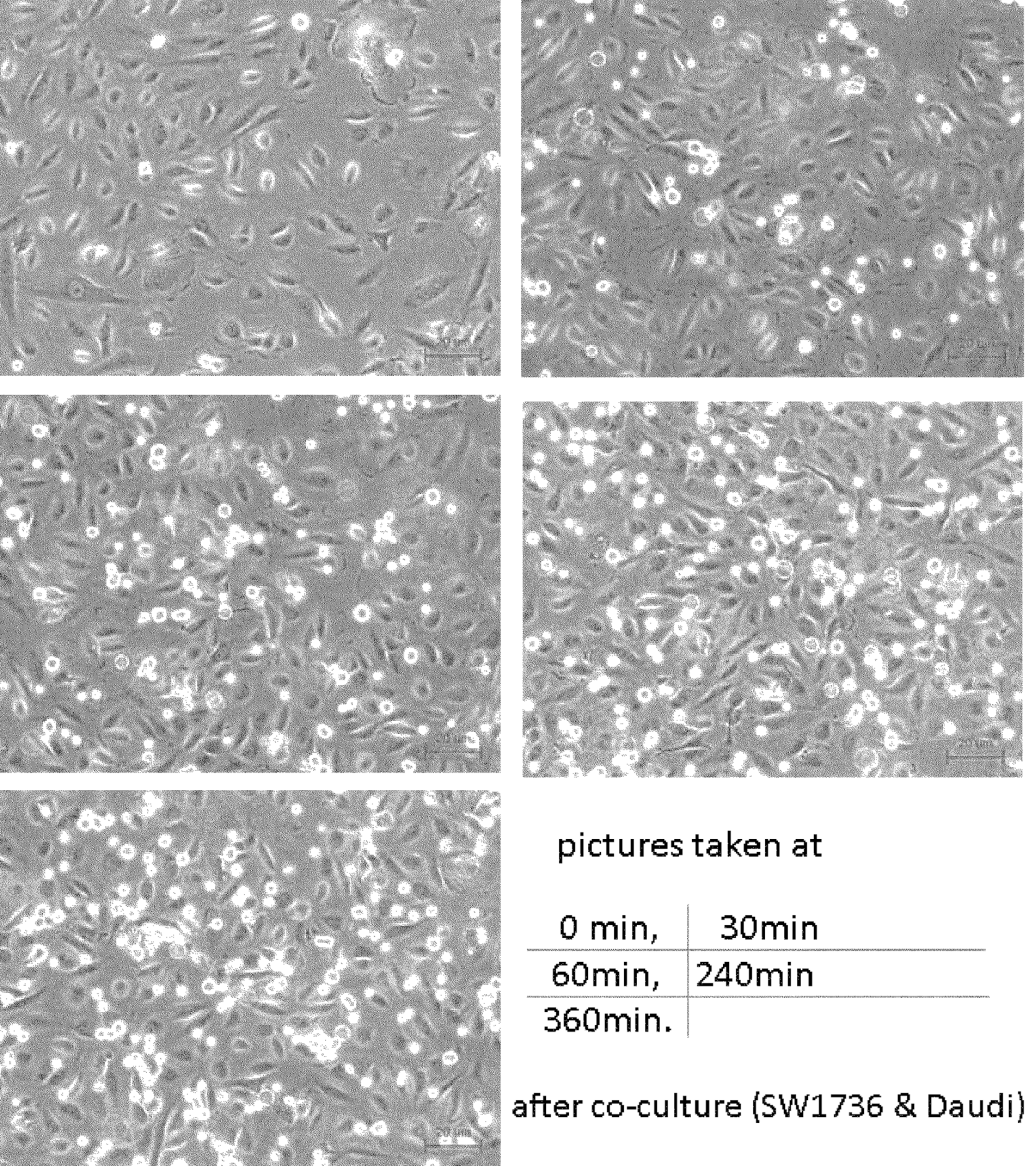
Co-cultured Daudi and SW1736 Cell Lines

SW1736 was revealed as a spindle shaped, adherent cell, meanwhile the Daudi was a round shaped, floating cell. After co-culture, the lymphocytic Daudi cells had a strong combined tendency to SW1736 thyroid carcinoma cells with a temporal course (Figure 5). 

**Figure 6.**
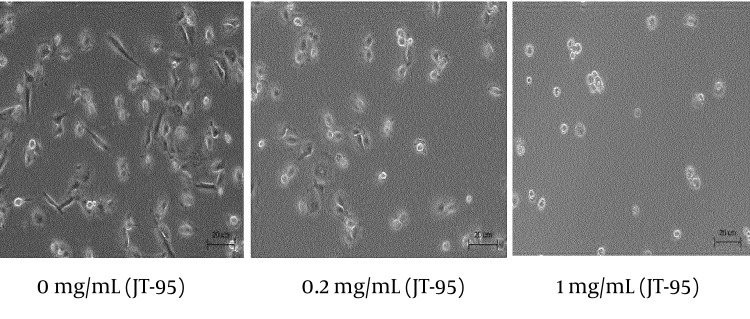
Co-cultured Daudi and SW1736 Cell Lines With JT-95

In contrast, combined cells were decreased, with an increase of pretreated JT-95 concentrations (Figure 6). 

**Figure 7.**
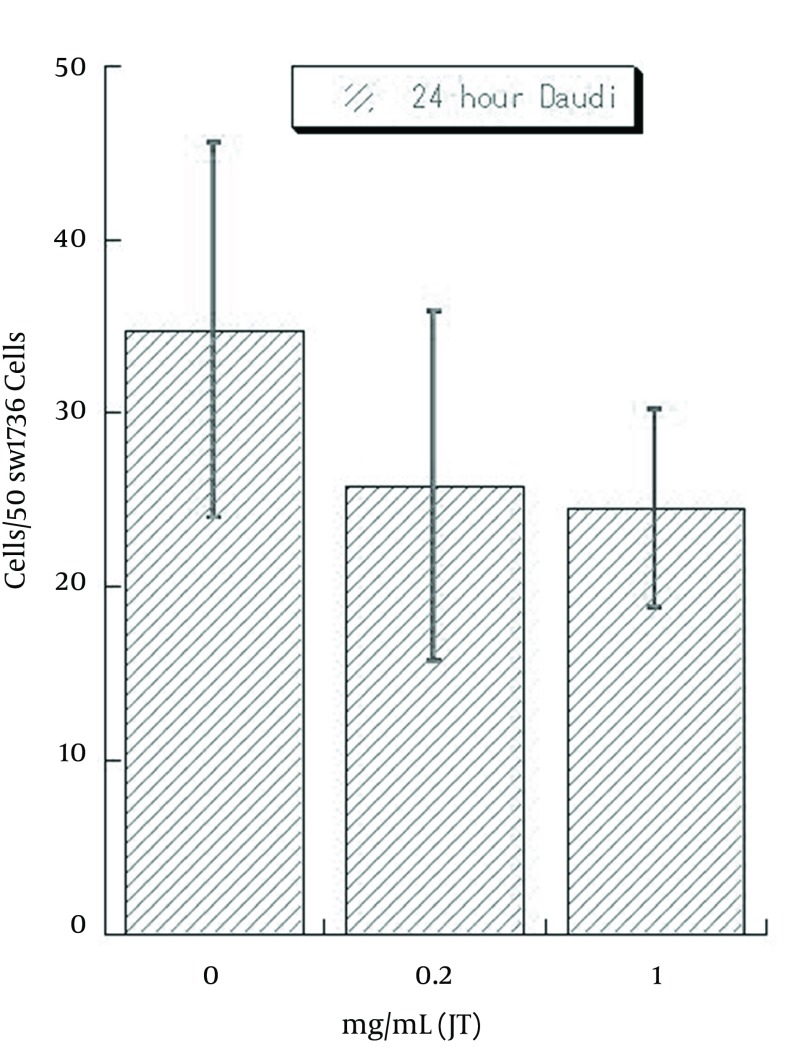
Numbers of Combined Daudi Cells on 50 SW1736 Cells at JT-95 Pretreated Status

As Daudi were floating cells, un-attached Daudi cells were washed out after 24 hours of co-incubation, the number of combined Daudi cells on 50 SW1736 cells were counted under a phase contrast microscope. The average number of cells in each of the 6 wells were 35.2/50 cells (range 25-46) with 0 mg/mL of JT-95, 25.3/50 cells (range 15-36) with 0.2 mg/mL, 22.2/50 cells (range 18-31) with 1 mg/mL, respectively (Figure 7). 

## 5. Discussion

Many studies have attempted to improve thyroid tumors diagnosis. FN in the cytoplasm of thyroid follicular cells is associated with transformation, and the co-expression of sFN, GAL3 and HBME1 is commonly observed in carcinomas, but it is restricted in benign lesions (12, 13). Moreover, FN is alternatively spliced, leading to isoforms such as the extracellular domains A/B and III-CS, especially during the tumor genesis. These uniquely glycosylated isoforms (III-CS) are predominantly expressed by fetal and neoplastic cells and have been designated as OnfFN (14). OnfFN has a wide spectrum of tumor specificity includes; hepatocellular carcinoma, breast carcinoma, and sarcoma cell lines (VA13) (15). OnfFN molecular weight (MW) is recognized as 310 - 335 kDalton (14-16). OnfFN is successful in detecting PTC and FTC using the RT-PCR method regarding thyroid carcinoma (7-9). The MoAb designated JT-95, which reacts with an antigen in thyroid carcinoma, has been produced with membrane fractions of human PTC. For other organic carcinomas, JT-95 responds to pancreatic and ovarian carcinomas, but it does not react with hepatocellular carcinoma or breast carcinomas. The antigen detected by JT-95 in thyroid carcinomas has an apparent MW of 250 kDalton (10).

Antigenic analysis of JT-95 revealed the presence of two antigens, glycosylated FN including sialic acid, and glycosylated gangliosides (11). Taking into account tumor specificity, differences in molecular size, and the two antigens, we believe that the epitope structure of JT-95 is distinct from that of OnfFN. Recently, it is thought that EMT would occur in carcinoma cells at the first stage of metastasis or invasion, by transforming growth factor β (TGF-β), or turning on the retinoblastoma (Rb) gene (17, 18). Depletion of the Rb gene induces down-regulation of the adhesion molecule E-cadherin, which triggers EMT (19). The quantity of cadherin between cell to cell is decreased and ECM molecules are abundantly secreted in carcinoma cells as EMT is induced, and the carcinoma cell moves easily in tissues with the co-action of ECM and integrin - FAK (focal adhesion kinase) (20). Moreover, the inactivation of the Rb gene also induces up-regulation of ZEB expression and induction of an invasive phenotype in breast carcinoma and thyroid papillary carcinoma (21). In the alternative model, differentiated epithelial cells are transformed during a process of EMT and acquire cancer stem cell (CSC)-like phenotype. This notion is supported by the fact that gene expression patterns in EMT-induced cells are nearly identical to those in CSCs.

CSCs have self-renewal activity and lead to metastasis of cancer cells and construct a lesion pathologically similar to the primary tumor in distant organs (22). In this study, we hypothesized that secretion of sFN in thyroid carcinoma cells’ ECM were caused by EMT, due to the fact that sFN was detected only in thyroid carcinoma cells, but not in normal thyroid cells and connective tissues. On the other hand, the increasing amount of sFN detected by JT-95 correlated to the lymph node metastasis in thyroid carcinomas. There are many papillary carcinomas of score>5 compared to follicular carcinoma. This accords with the fact that papillary carcinoma has more lymph node metastasis than follicular carcinoma clinically (4-6). In addition, B and T lymphocytes, components of the lymph nodes, hold integrin-α on their cell surface as fibronectin receptors (23, 24).

Therefore, we suggest that the coactions of sFN and integrin-α are possible causes of lymph node metastasis in thyroid malignancies. The administration of JT-95 blocked adhesions between Daudi and SW1736 cells supports the hypothesis in our ideas. However, the interaction between sFN and integrin is only one of the inhibitory possibilities. We think that further investigation is needed as JT-95 may have a therapeutic effect by inhibiting the attachment CBCs, which have the ability to reproduce in lymph nodes, and they could exist in EMT cells. 

In conclusion, increasing the number of cells with sFN expression in thyroid tumors is correlated with lymph node metastasis, which suggests the malignancy of thyroid tumors. It may be possible to utilize JT-95 as a therapeutic agent to prevent lymph node metastasis.
